# Chest CT Imaging Features of Typical Covert COVID-19 Cases

**DOI:** 10.7150/ijms.48614

**Published:** 2021-03-15

**Authors:** Meng Dai, Liu Ouyang, Fan Yang, Heshui Shi, Jiazheng Wang, Xiaoyu Han, Osamah Alwalid, Yukun Cao, Dan Yang, Yuwen Li, Wenying Zhu, Jie Liu

**Affiliations:** 1 Department of Radiology, Union Hospital, Tongji Medical College, Huazhong University of Science and Technology, Wuhan 430022, China.; 2 Hubei Province Key Laboratory of Molecular Imaging, Wuhan 430022, China.; 3 Department of Orthopaedics, Union Hospital, Tongji Medical College, Huazhong University of Science and Technology, Wuhan 430022, China.; 4 MSC Clinical & Technical Solutions, Philips Healthcare, Beijing 100000, China.; 5 Department of Respiratory and Critical Care Medicine, Union Hospital, Tongji Medical College, Huazhong University of Science and Technology, Wuhan 430022, China.; 6 Hayward Genetics Center, Tulane University School of Medicine, New Orleans, Louisiana, USA.

**Keywords:** RT-PCR, GGO, COVID-19, CT, covert coronavirus infections.

## Abstract

**Purpose:** To analyze the chest CT imaging findings of patients with initial negative RT-PCR and to compare with the CT findings of the same sets of patients when the RT-PCR turned positive for SARS-CoV-2 a few days later.

**Materials and methods:** A total of 32 patients (8 males and 24 females; 52.9±7years old) with COVID-19 from 27 January and 26 February 2020 were enrolled in this retrospective study. Clinical and radiological characteristics were analyzed.

**Results:** The median period (25%, 75%) between initial symptoms and the first chest CT, the initial negative RT-PCR, the second CT and the positive RT-PCR were 7(4.25,11.75), 7(5,10.75), 15(11,23) and 14(10,22) days, respectively. Ground glass opacities was the most frequent CT findings at both the first and second CTs. Consolidation was more frequently observed on lower lobes, and more frequently detected during the second CT (64.0%) with positive RT-PCR than the first CT with initial negative RT-PCR (53.1%). The median of total lung severity score and the number of lobes affected had significant difference between twice chest CT (P=0.007 and P=0.011, respectively).

**Conclusion:** In the first week of disease course, CT was sensitive to the COVID-19 with initial negative RT-PCR. Throat swab test turned positive while chest CT mostly demonstrated progression.

## Introduction

Since December 2019, several cases of pneumonia of unknown etiology have been reported in Wuhan, Hubei Province of China 1-4. A novel coronavirus was identified by the Chinese Center for Disease Control and Prevention (CDC) from the throat swab samples 2 and then was named as severe acute respiratory syndrome coronavirus 2 (SARS-CoV-2) on 11 February by World Health Organization (WHO)^5^. Now the new coronavirus outbreak has caused global infection of 57,639,631people by 22 November 2020, while the number is fast increasing particularly in Europe and the United States 6. As the virus spreads rapidly, more and more researchers are now concerned about patients with mild or no symptoms ("hidden infections") who might be transmitting the virus to others 7-9. Scientists suggested that these covert cases could represent 60% of all infections and could be seeding new outbreaks 7.

It is essential to detect these covert coronavirus infections at the early stage, and real time reverse-transcription-polymerase chain-reaction (RT-PCR) is routinely used to detect pathogenic viruses from respiratory secretions 10. However, studies found that some patients with SARS-CoV-2 infection had initial negative RT-PCR but positive chest CT, and the RT-PCR turned positive several days later 11-16. Xingzhi Xie et al. 11 and Yicheng Fang et al. 12 first reported 5 /167 (2.9%) and 15/51 (29.4%) patients with negative RT-PCR and positive CT consistent with coronavirus disease 2019 (COVID-19) at initial presentation, respectively. Tao Ai et al. 14 and Herpe et al. 16 found that 53% to 93% of patients had initial positive chest CT before the positive RT-PCR results in the studies with large sample participants.

Patients with initial negative RT-PCR may be covert infections who may not be identified and consequently could continue to spread this disease and chest CT scans are emerging as a useful tool in the screening of COVID-19. High sensitivity (67-100%) and relatively low specificity (25-90%) was reported for the CT scans, while the sensitivity of RT-PCR was reported to be modest (53-88%) 17-19. The clinical and radiological manifestations in COVID-19 cases with negative RT-PCR results have been preliminarily discovered by Guanjing Lang et al. 15, which were just a briefly description without statistical analysis due to small sample size (8 patients). Furthermore, it remains unclear about the features of the same set of patients with COVID-19 at the two periods of initial negative and then positive RT-PCR results.

In our study, for each patient with COVID-19, the synchronized chest CT scans were performed in each period (two periods were defined based on RT-PCR results: initial negative RT-PCR results, subsequent positive RT-PCR results) and corresponding RT-PCR tests were performed within 3 days as chest CT. The purpose of this paper was to analyze the serial chest CT and RT-PCR findings in patients with COVID-19 and to investigate how the chest CT changes differ between the same set of patients with negative and subsequent positive RT-PCR results. It was hypothesized that imaging features of early chest CT may facilitate an early diagnosis of suspected patients especially with mild or no symptoms.

## Materials and Methods

This retrospective study was approved by Institutional Review Board (IRB). Informed consent for this retrospective study was waived.

### Patients

In total 539 consecutive COVID-19 patients, who were confirmed by real-time RT-PCR test and who went through serial chest CT scans following their admission to isolation ward of Union Hospital (Wuhan, China) and Jianghan Shelter Hospital between 27 January 2020 and 26 February 2020, were reviewed for this retrospective study. All these COVID-19 patients have been confirmed and monitored by serial chest CT scans and real-time RT-PCR tests on respiratory secretions collected by the throat swab test. For patients with multiple RT-PCR assays, the diagnosis of COVID-19 was confirmed when any one of the nucleic acid test results was positive. Typical imaging findings associated with COVID-19 infection, including ground-glass opacity (GGO) and/or mixed GGO and mixed consolidation 20, 21, were considered as positive CT.

**Figure 1:**
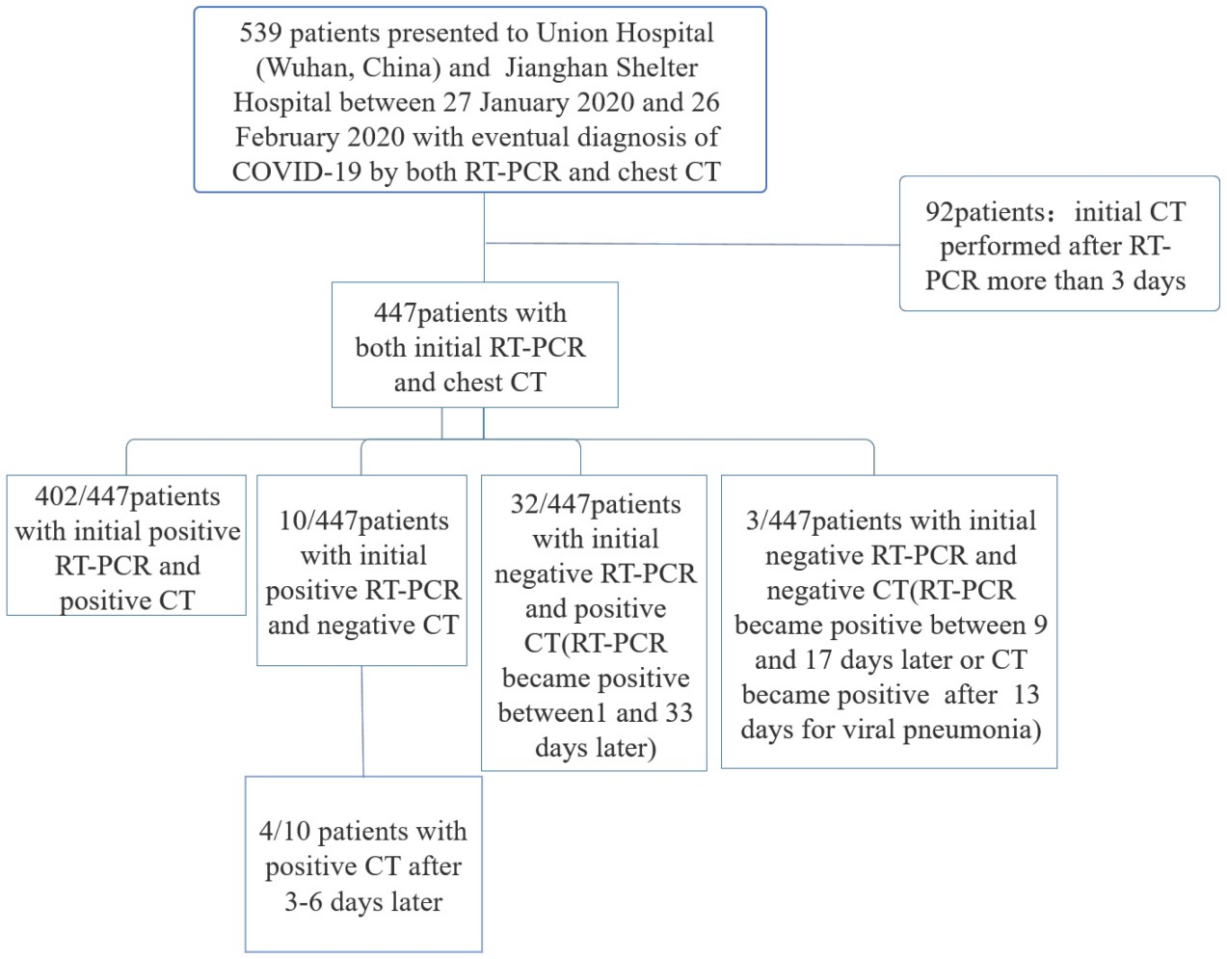
Flowchart of this study.

Patients were excluded if the initial chest CT was performed more than 3 days after initial RT-PCR** (Figure 1)**. Repeated positive RT-PCR testing after the initial RT-PCR was used to analyze conversion of RT-PCR results, in correlation with the chest CT scans. 32 (8 males and 24 females, 29-82 years old) out of these 539 patients initially returned with negative RT-PCR results and positive CT findings, whose RT-PCR were confirmed positive by a later repeat test.

### CT image acquisition

Chest CT scans were acquired in supine position by three commercial multi-detector CT scanners (Philips Ingenuity Core128, Philips Medical Systems, the Netherlands; GE Discovery CT750 HD, General Electric Company, the USA; TOSHIBA Activion16, TOSHIBA CORPORATION, Japan). To minimize motion artifacts, patients were instructed on breath-holding; CT images were then acquired during a single breath-hold. For CT acquisition, the tube voltage was 120kVp with automatic tube current modulation. From the raw data, CT images were reconstructed into a matrix size of 512 × 512 (thickness of 1.5mm and increment of 1.5mm) in transverse orientation. The scan ranged from the level of the upper thoracic inlet to the inferior level of the costophrenic angle.

### Chest CT evaluation

The international standard nomenclature, defined by the Fleischner Society Glossary and the peer-reviewed literature investigating viral pneumonia, was utilized to describe the major CT findings 22-24. The chest CT image was evaluated for the following characteristics for each of the 32 patients: (1) ground glass opacity (GGO), (2) consolidation, (3) number of lobes affected by GGO or consolidation, (4) other interstitial abnormalities (e.g. reticulation, interlobular septal thickening, crazy-paving pattern), (5) underlying lung diseases (e.g. emphysema or fibrosis), (6) pleural effusion, (7) mediastinal lymphadenopathy (defined as lymph node size ≥10 mm in short-axis dimension), and (8) other abnormalities (e.g., cavitation, calcification, and bronchiectasis).

The pulmonary involvement was evaluated semi-quantitatively for each lung lobes 25. According to the size of pulmonary involvement, a score was given to each of the five lung lobes by visual evaluation of the CT scans. Score 0, 1, 2, 3, 4, and 5 were defined by 0%, <5%, 25%, 26~49%, 50~75%, and >75% pulmonary involvement, respectively. The total CT score was calculated as the summation of the scores of all five individual lobes, ranging from 0~25, which represent the pulmonary involvement from none to maximum. The focal distribution of pulmonary abnormalities was documented as well.

All CT images were initially analyzed by two radiologists (M.D and J.L, who had 5 and 8 years of experience in thoracic radiology, respectively) using the institutional digital database system (Vue PACS, version 11.3.5.8902, Carestream Health, Canada) without access to clinical and laboratory findings. Images were reviewed independently, and final decisions were reached by discussion and consensus.

### Effective Dose Calculation

A record of the dose-length product (DLP) for each CT scan performed was recorded. The DLP values, along with DLP conversion coefficients (k) were used to estimate the effective dose received with each respective CT scan, K=0.014 mSv ·mGy ^-1^ ·cm ^-1^ 26, 27.

### Statistical analysis

Wilcoxon signed rank test and McNemar-bowker test were used for the difference between the first CT and second CT findings in patients who had twice Chest CTs and corresponding RT-PCR tests and whose twice RT-PCR tests were ≤14 days apart. Number of lobes affected and total lung severity score were presented as median (25%, 75%). A P value of < 0.05 was considered to be statistically significant. Statistical analyses were performed using SPSS Statistics (SPSS, version 22, IBM, Chicago, IL, USA).

## Results

### Patient Groups

539 patients with the laboratory-confirmed COVID-19 pneumonia were admitted to Wuhan Union Hospital and Jianghan Shelter Hospital between 27 January 2020 and 26 February 2020. There were 402 (74.6%) patients with initial positive RT-PCR and positive CT results for viral pneumonia, 10 (1.9%) patients with positive initial RT-PCR but negative initial CT, 32(5.9%) patients with positive initial CT but negative initial RT-PCR, and 3(0.6%) patients with negative initial RT-PCR and negative initial CT.** (Figure 1).**

### Clinical Characteristics

A total of 32 patients (including 8 males and 24 females, with the age of 52.9±7years) with negative initial RT-PCR and positive CT were enrolled for further analysis. Fever (24/32[75.0%]) and cough (18/32[56.3%]) were the most common initial symptoms, while 2(6.3%) patients had no obvious symptoms** (Table 1).**

The median period (25%, 75%) between initial symptoms and the first chest CT, the initial negative RT-PCR were 7(4.25, 11.75) and 7(5, 10.75), respectively. The median period between initial symptoms and the second chest CT, the positive RT-PCR were 15(11, 23) and 14(10, 22), respectively** (Table 1).**

**Figure 2:**
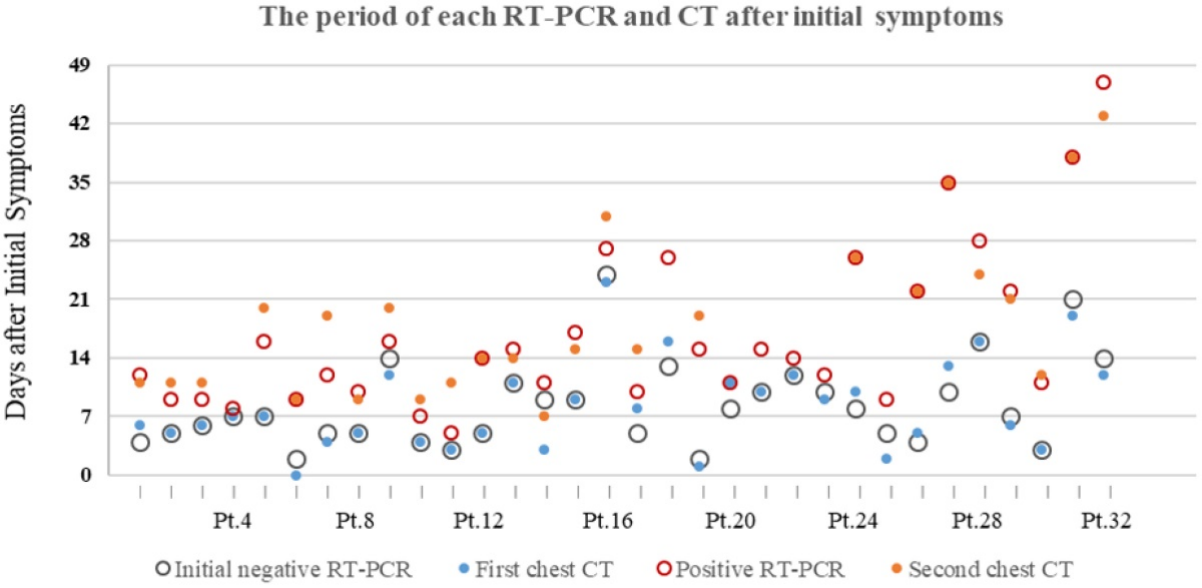
** The period of each RT-PCR and chest CT examinations after initial symptoms of 32 patients with COVID-19.** Pt.=Patients.

16/32 (50%) patients had first chest CT and initial RT-PCR performed within a week after initial symptoms onset, resulting in positive CT and negative RT-PCR results. The other 16/32 (50%) patients were CT-positive between 8 and 23 days after initial symptoms, while RT-PCR result was initially negative between 8 and 24 days. The majority of patients (17/32[53.1%]) were presented positive RT-PCR results within two weeks after initial symptoms onset. 6/32 (18.8%) and 6/32 (18.8%) patients turned positive for RT-PCR test in the 3^nd^ ,4^th^ week, respectively. 3/32 (9.4%) patients had positive RT-PCR result after 4 weeks later after initial symptoms onset, the longest of which is 47 days.** (Figure 2)**.

### Chest CT Evaluation

**Figure 3:**
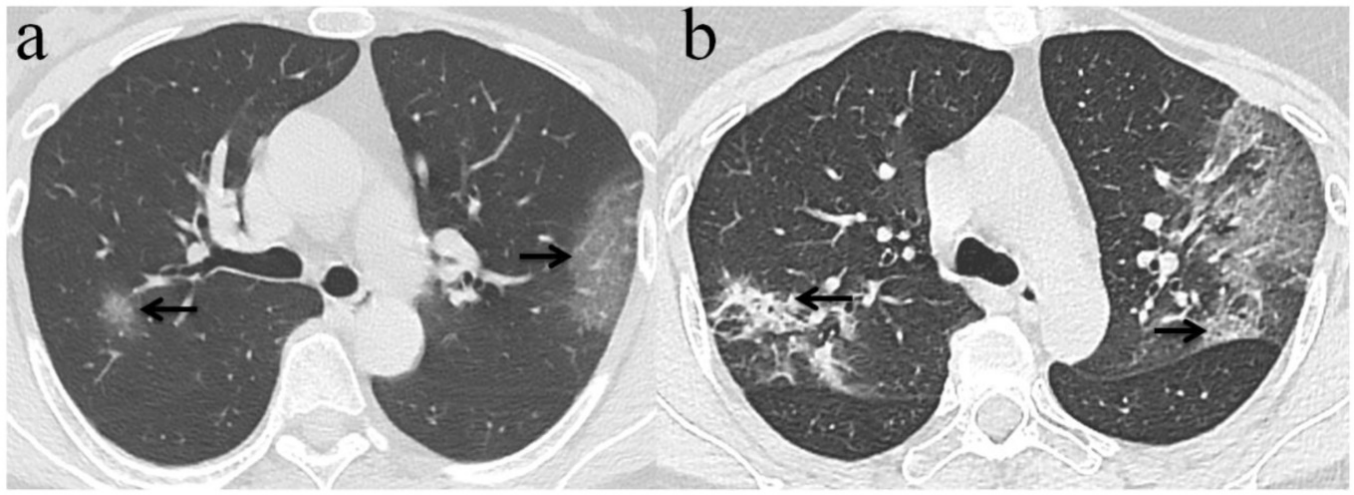
** Chest CT imaging findings of COVID-19 pneumonia in a 56-year-old female patient presenting with fever (range:38.1-39℃) for five days.** (a) GGO was presented in the upper lobes of bilateral lung with initial negative RT-PCR (arrow); (b) 6 days later, the upper lobes of bilateral lung show extensive consolidation and GGO while RT-PCR test turned positive. (arrow).

**Figure 4:**
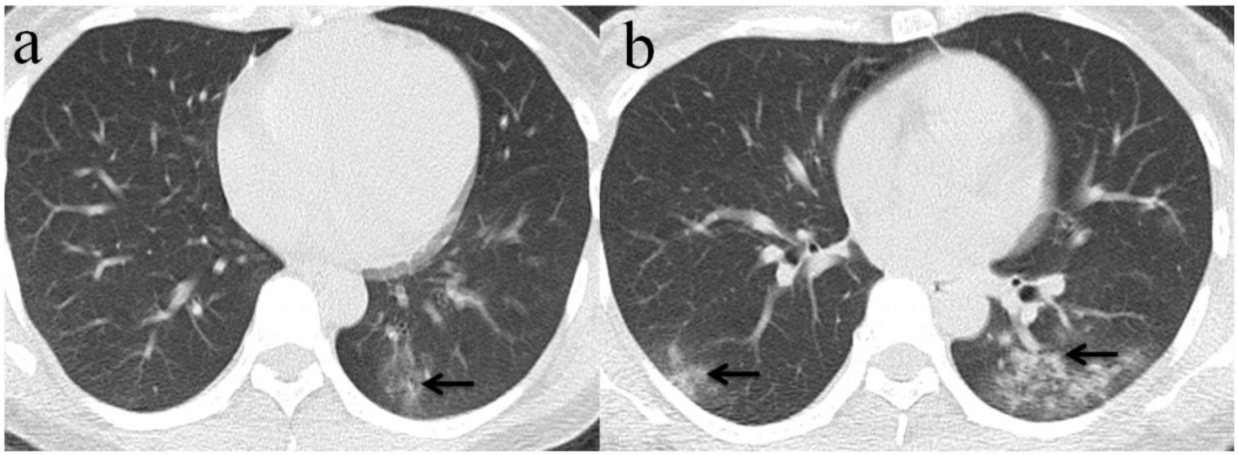
** Chest CT imaging findings of COVID-19 pneumonia in a 30-year-old female patient presenting with fever (range:38.1-39℃) for four days.** (a) Subpleural GGO was presented in the left lower lobe with initial negative RT-PCR (arrow);(b) 5 days later, there was an enlarged region of GGO and consolidation(arrow) presented in lower lobes of bilateral lung while RT-PCR test turned positive. (arrow).

**Figure 5:**
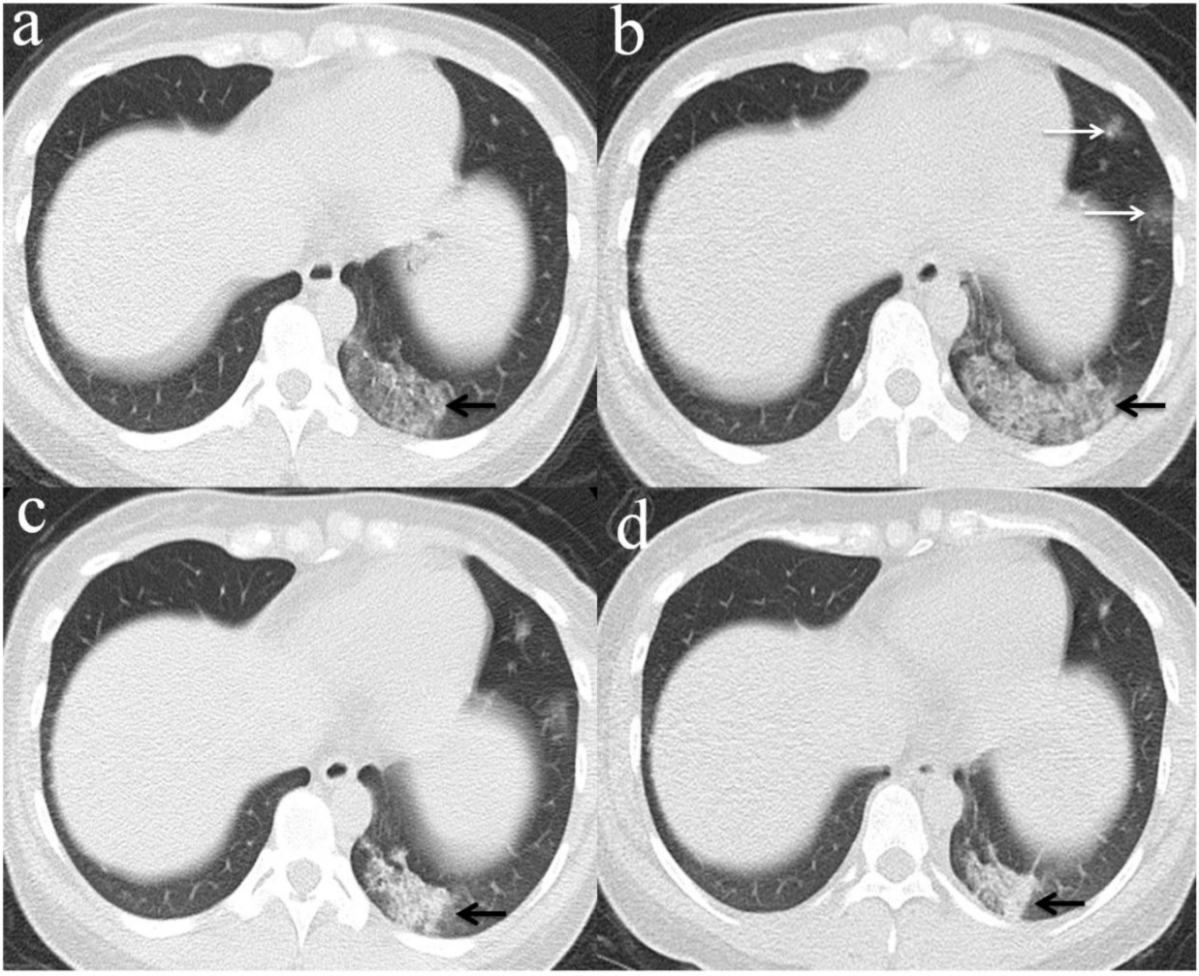
** Evolution of chest CT imaging findings in a 30-year-old female patient presenting with fever (range:38.1-39℃), cough and expectoration for four days.** (a)6 days after initial symptoms, subpleural GGO with partial consolidation was presented in the left lower lobe(black arrow).While, the patient's initial RT-PCR was negative; (b) day 11, there was an enlarged region of GGO and consolidation(black arrow),and was demonstrated new GGO appeared in other lesions of left lobe(white arrows).One day later, swab test turned positive for SARS-CoV-2; (c) day 16, the overall range of lesions was smaller than that of day 11, but the subpleural consolidation were more frequent(black arrow);(d) day 22, continued resolution with consolidation(black arrow) and other lesions of left lobe demonstrated remission.

Among the 32 patients with negative initial RT-PCR and first positive CT, only 25 patients had the second chest CT when RT-PCR test turned positive. GGO was the most frequent CT findings at the first and second chest CT [96.9% (31/32) and 84% (21/25), respectively]** (Figure 3)**, and the right upper lobe (22/32[68.8%]) was the most commonly affected at initial chest CT scan with negative RT-PCR. Consolidation was more frequently observed on the lower lobes, and more frequently detected during the second CT (16/25[64.0%]) with positive RT-PCR than the first CT with initial negative RT-PCR (17/32[53.1%])** (Table 2, Figure 3, 4)**. More than two affected lung lobes were detected in 65.6% (21/32) patients at the first chest CT, and this ratio went up to 84.0% (21/25) at the second chest CT, while 12/25 (48.0%) patients had all five lobes affected. Bilateral lung involvement was more often than unilateral both at the first (22/32[68.8%]) and second (22/25[88.0%]) chest CT **(Table 2)**. Subpleural distribution characterized the lung involvement in most patients (24/32[75.0%]) at the first chest CT, which presented in 11/25 (44%) patients at the second CT **(Figure 5)**. However, the crazy-paving pattern is not as common at the first (4/32[12.5%]) and second (3/25[12.0%]) chest CT. No patients had cavitation, calcification, or lymphadenopathy.

**Figure 6:**
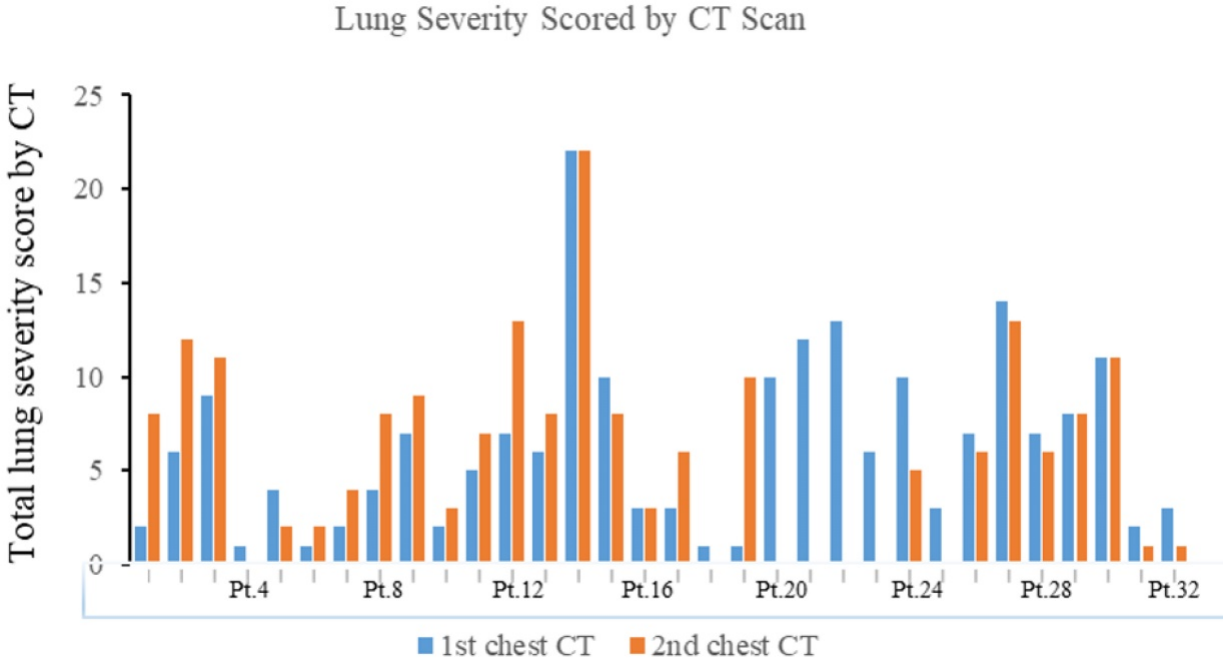
** The total lung severity score in the first and second chest CT of 32 patients with COVID-19.** Pt.=Patients.

The median total lung severity score was 6 in the initial CT examination and 8 in the second chest CT **(Table 2, Figure 6)**. Noticeably, 13/25 (52%) patients progressed mildly, 8/25 (32%) patients demonstrated remission, and the other 4/25 (16%) patients remained unchanged appearance in the second chest CT.

The median effective doses for the first and second chest CTs were 3.48 (2.75, 4.08) and 2.9 (2.43, 3.69), respectively.

### Comparison of CT findings in patients at different RT-PCR time

There are 25 patients who had twice Chest CTs and corresponding RT-PCR tests and 6 patients were excluded who had twice RT-PCR tests >14 days apart because of the short supply of kits. Among the other 19 patients, the median of total lung severity score and the number of lobes affected had significant difference between the first CT with initial negative RT-PCR and the second CT with positive RT-PCR (P=0.007 and P=0.011, respectively) **(Table 3)**.

## Discussion

Our study revealed that in the first week of disease course, CT was sensitive to the COVID-19 with initial negative RT-PCR, and subsequent RT-PCR result turned positive while chest CT mostly demonstrated progression including more lung lobes involvement and higher severity score. RT-PCR has been used as the gold standard so far for COVID-19 diagnosis 10, but this test does have false negatives 17-19. Patients with initial negative RT-PCR could infect other people especially when Chest CT indicated positive who could be covert infection. In our study, most of the patients initially presented with negative RT-PCR within one week after initial symptoms, which frequently turned positive in the second week accompanied by progressive findings on the second chest CT. This may be explained by a report from Peiris JSM et al. 28 where quantitative RT-PCR of nasopharyngeal aspirates in patients infected with SARS showed viral loads peaking approximately 10 days after symptom onset. The increase in viral load to a detectable level by RT-PCR in the second week was evidenced by the progressive lesion findings in the second chest CT.

Chest CT is a key component in the early diagnosis of patients with COVID-19. Our research had demonstrated several CT imaging features of the patients at initial negative RT-PCR, where GGO of the right upper lobe was most common (22/32, 68.8%), different to a report by Chung M et al. 21, where the right lower lobe was more frequently involved (16/21, 76%). Consolidation was more frequently detected on the second chest CT with positive RT-PCR than the first CT in our study, in consistency with the findings reported by Huang P et al. 13. Pan F et al. 20 demonstrated that lung involvement evolved to consolidation within two weeks after symptom onset. Similarly, in our study, consolidations also increased in distribution and extent on the second chest CT mostly acquired in the second week. Consistent with other studies 20, 21, subpleural distribution of lung lesion was also observed in our study on the initial chest CT, which was, however, less obvious in the second CT. Crazy-paving pattern was one of the most frequent CT findings in mild COVID-19 pneumonia in previous research 20. However, the proportion of crazy-paving pattern was not much high in both the first and second CTs in our cohort of the 32 patients.

Our study showed that the median of total lung severity score and the number of lobes affected had significant differences between the second chest CT with positive RT-PCR [8 (4, 11) and 5 (3, 5), respectively] and the first CT with initial negative RT-PCR [5(2,7) and 3(1,4), respectively], which reflected throat swab test turned positive while chest CT mostly demonstrated progression. Besides, the severity and progression course of lung changes on chest CT in our study were similar to the patients with mild COVID-19 pneumonia 20, 21, 29. Therefore, we may speculate that the COVID-19 infected patients with initial negative RT-PCR are generally of mild disease. The most common symptom in these patients with initial negative RT-PCR was fever, Consistent with previous study 15. Guanjing Lang et al. 15 reported 1/8 (12.5%) patient was asymptomatic, while 2/32 (6.3%) patients were asymptomatic in our study. We also found that in clinical practice, diagnosis of COVID-19 was sometimes made without any positive RT-PCR tests but typical chest CT findings and clinical evolution, since only one RT-PCR assay was performed in some patients, which was in broad agreement with other surveys 16.

The median effective doses for the first and second chest CTs were less than the limits provided by International Commission on Radiological Protection (ICRP) and National Committee on Radiological Protection (NCRP), where the recommended permissible annual dose was 20 mSv and 50 mSv, respectively 26.

This study has some limitations. First, separations and statistical comparisons in patients symptomatic or non-symptomatic have not been done here because the sample size was small. Second, the exposure history was not taken into separations and analysis because the majority of patients with COVID-19 had no accurate and reliable history of exposure in this retrospective study. Third, the CT scans for the included patients had different time intervals from the date of being infected.

In conclusion, we suggested the high sensitivity of CT in COVID-19 detection when compared to RT-PCR in the first week from symptom onset. In order to have a successful and efficient control of viral outbreak, the covert cases with initial negative RT-PCR results but characteristic radiographic features, including GGO, unilateral right upper lobe involvement, and subpleural distribution, should be isolated and repeat RT-PCR test to prevent further transmission of the virus to family and community, especially when they have epidemic history and related symptoms such as fever and cough. When throat swab test turned positive for SARS-CoV-2, chest CT mostly demonstrated progression, including increasing consolidations, more frequent bilateral lung involvement and higher severity score of lung involvement. A combination of repeated RT-PCR and CT scanning may be helpful for individuals with clinical suspicion of covert infection.

## Figures and Tables

**Table 1:** Cohort information of 32 patients with COVID-19.

Initial symptoms	Number (%)
Fever	24(75.0%)
37.3-38.0℃	9
38.1-39.0℃	11
>39℃	4
Cough	18(56.3%)
Expectoration	5(15.6%)
Chills	2(6.3%)
Dyspnea	3(9.4%)
Myalgia	5(15.6%)
Fatigue	7(21.9%)
Loss of appetite	15(46.9%)
Diarrhoea	7(21.9%)
Itching of eye	1(3.1%)
No symptoms	2(6.3%)
**The period of each RT-PCR and CT after initial symptoms**	**Median (25%,75%)**
The period between initial symptoms and the first chest CT(d)	7(4.25,11.75)
The period between initial symptoms and the second chest CT(d)	15(11,23)
The period between initial symptoms and initial negative RT-PCR(d)	7(5,10.75)
The period between initial symptoms and positive RT-PCR(d)	14(10,22)

**Table 2:** Evaluation of CT imaging distribution and severity of two periods for initial negative RT-PCR and positive RT-PCR of patients with COVID-19. Number (%).

	First chest CT(32 patients)	Second chest CT(25 patients)
**Ground glass opacities**	31(96.9%)	21(84.0%)
Right upper lobe	22(68.8%)	15(60.0%)
Right middle lobe	13(40.6%)	9(36.0%)
Right lower lobe	18(56.3%)	16(64.0%)
Left upper lobe	15(46.9%)	16(64.0%)
Left lower lobe	19(59.4%)	16(64.0%)
**Consolidation**	17(53.1%)	16(64.0%)
Right upper lobe	2(6.3%)	6(24.0%)
Right middle lobe	2(6.3%)	4(16.0%)
Right lower lobe	12(37.5%)	13(52.0%)
Left upper lobe	4(12.5%)	6(24.0%)
Left lower lobe	13(40.6%)	12(48.0%)
**Number of lobes affected**		
1.0	9(28.1%)	4(16.0%)
2.0	1(3.1%)	1(4.0%)
3.0	8(25.0%)	4(16.0%)
4.0	5(15.6%)	4(16.0%)
5.0	9(28.1%)	12(48.0%)
Unilateral lung	10(31.3%)	3(12.0%)
Bilateral lung	22(68.8%)	22(88.0%)
More than 2 lobes affected	21(65.6%)	21(84.0%)
**Median of total Lung Severity Score (25%,75%)**	6(2.25,9.75)	8(3.5,10.5)
**Crazy-paving pattern**	4(12.5%)	3(12.0%)
**Peripheral distribution**	24(75.0%)	11(44.0%)
**Pleural effusion**	0(0%)	1(4.0%)
**Other abnormalities**		
Cavitation	0(0%)	0(0%)
Interlobular septal thickening	6(18.8%)	4(16.0%)
Calcification	0(0%)	0(0%)
Lymphadenopathy	0(0%)	0(0%)
Dose Length Product(mGy.cm)	248 (196,291)	207(174,263)
Effective Dose(mSv)	3.48 (2.75,4.08)	2.9(2.43,3.69)

**Table 3:** Comparison of CT findings in patients who had twice Chest CTs and corresponding RT-PCR tests and whose twice RT-PCR tests were ≤14 days apart.

	First chest CT( 19 patients)	Second chest CT(19 patients)	P value
**Number of lobes affected**	3(1,4)	5(3,5)	0.011*
Bilateral lung	13(68.4%)	17(89.5%)	0.125
More than 2 lobes affected	13(68.4%)	16(84.2%)	0.375
**Median of total Lung Severity Score (25%,75%)**	5(2,7)	8(4,11)	0.007*
**Crazy-paving pattern**	3(15.8%)	3(15.8%)	1.000
**Peripheral distribution**	16(84.2%)	13(68.4%)	0.375

**Note**: Data are median (IQR), n (%). p values comparing first Chest CT and second Chest CT results are from the McNemar-bowker test. *p values comparing first Chest CT and second Chest CT results are from Wilcoxon signed rank test, P value < 0.05.

## Abbreviations

RT-PCR: reverse-transcription-polymerase chain-reaction
GGO: Ground glass opacities
SARS-CoV-2: Severe Acute Respiratory Syndrome Coronavirus 2
COVID-19: coronavirus disease 2019
